# Basal cisternostomy for traumatic brain injury: A case report of unexpected good recovery

**DOI:** 10.1016/j.cjtee.2021.12.008

**Published:** 2021-12-28

**Authors:** Manuel De Jesus Encarnacion Ramirez, Rossi Evelyn Barrientos Castillo, Anton Vorobiev, Nikita Kiselev, Amaya Alvarez Aquino, Ibrahim E. Efe

**Affiliations:** a Department of Neurosurgery, Russian People's Friendship University, Moscow, Russia; b Department of Neurosurgery, Municipal Hospital, Podolsk, Russia; c Department of Neurosurgery, International Center for Neurological Restoration, Havana, Cuba; d Charité – Universitätsmedizin Berlin, Corporate Member of Freie Universität Berlin, Humboldt-Universität zu Berlin, And Berlin Institute of Health, Berlin, Germany

**Keywords:** Basal cisternostomy, Cerebral edema, Decompressive craniectomy, Neurotrauma, Traumatic brain injury

## Abstract

In subarachnoid hemorrhage following traumatic brain injury (TBI), the high intracisternal pressure drives the cerebrospinal fluid into the brain parenchyma, causing cerebral edema. Basal cisternostomy involves opening the basal cisterns to atmospheric pressure and draining cerebrospinal fluid in an attempt to reverse the edema. We describe a case of basal cisternostomy combined with decompressive craniectomy. A 35-year-old man with severe TBI following a road vehicle accident presented with acute subdural hematoma, Glasgow coma scale score of 6, fixed pupils and no corneal response. Opening of the basal cisterns and placement of a temporary cisternal drain led to immediate relaxation of the brain. The patient had a Glasgow coma scale score of 15 on postoperative day 6 and was discharged on day 10. We think basal cisternostomy is a feasible and effective procedure that should be considered in the management of TBI.

## Introduction

Severe traumatic brain injury (TBI) is a life-threatening condition, which continues to cause substantial morbidity and mortality worldwide. The pathogenesis of severe TBI includes a primary injury, which is directly related to the physical impact on the brain, and a delayed secondary injury due to metabolic and inflammatory cascades resulting in edema, ischemia, and intracranial hypertension.^1^

Cisternal opening is commonly used as part of cerebrovascular or skull base approaches to release cerebrospinal fluid (CSF) and relax the brain to allow easier access to a lesion. This technique has been proposed to reduce cerebral edema in TBI and has since gained increasing popularity.^2^, ^3^, ^4^

In brief, basal cisternostomy involves opening of the basal cistern via a pterional or subfrontal route. CSF is drained from both supratentorial and infratentorial cisterns to achieve brain relaxation, and a temporary drain is inserted into the basal cistern to facilitate postoperative CSF outflow.^5^

## Case report

### General condition

We here demonstrated the unanticipated recovery of a 35-year-old male patient who presented with a severe TBI following a traffic accident. We used the basal cisternostomy as an adjuvant treatment combined with decompressive craniectomy. To the best of our knowledge, this is the first report of TBI treated with basal cisternostomy in the Russian Federation.

The patient was brought to the emergency department of the Municipal Hospital in Podolsk. At the time of admission, the patient had a Glasgow coma scale (GCS) of 6/15. He was intubated, and had bilaterally fixed pupils of 4 mm and no corneal response. His heart tones were clear, rhythmic, and the blood pressure was 140/90 mmHg at a heart rate of 88 beats/min. Physical examination showed loss of continuity and anatomical deformity of the frontotemporal region and the right orbital region.

**Fig. 1:**
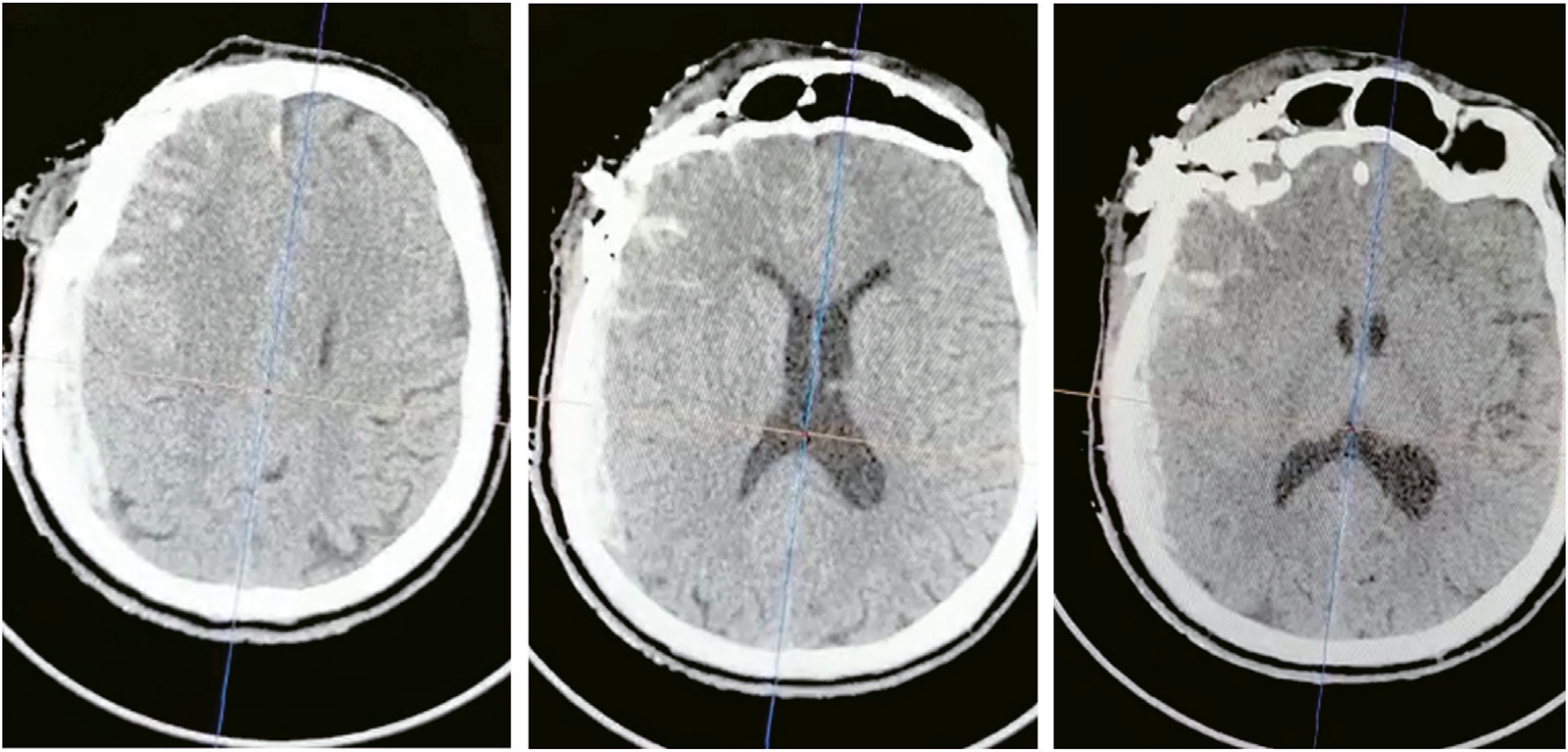
Preoperative axial CT showing right-sided subdural hematoma, effaced basal cisterns and midline shift.

Cranial CT showed a multi-comminuted fracture of the right skull base, pneumocephaly and an acute subdural hematoma of the right hemisphere causing a midline shift of 8 mm to the left side (Fig. 1). Immediate surgical intervention was indicated.

### Surgery

**Fig. 2:**
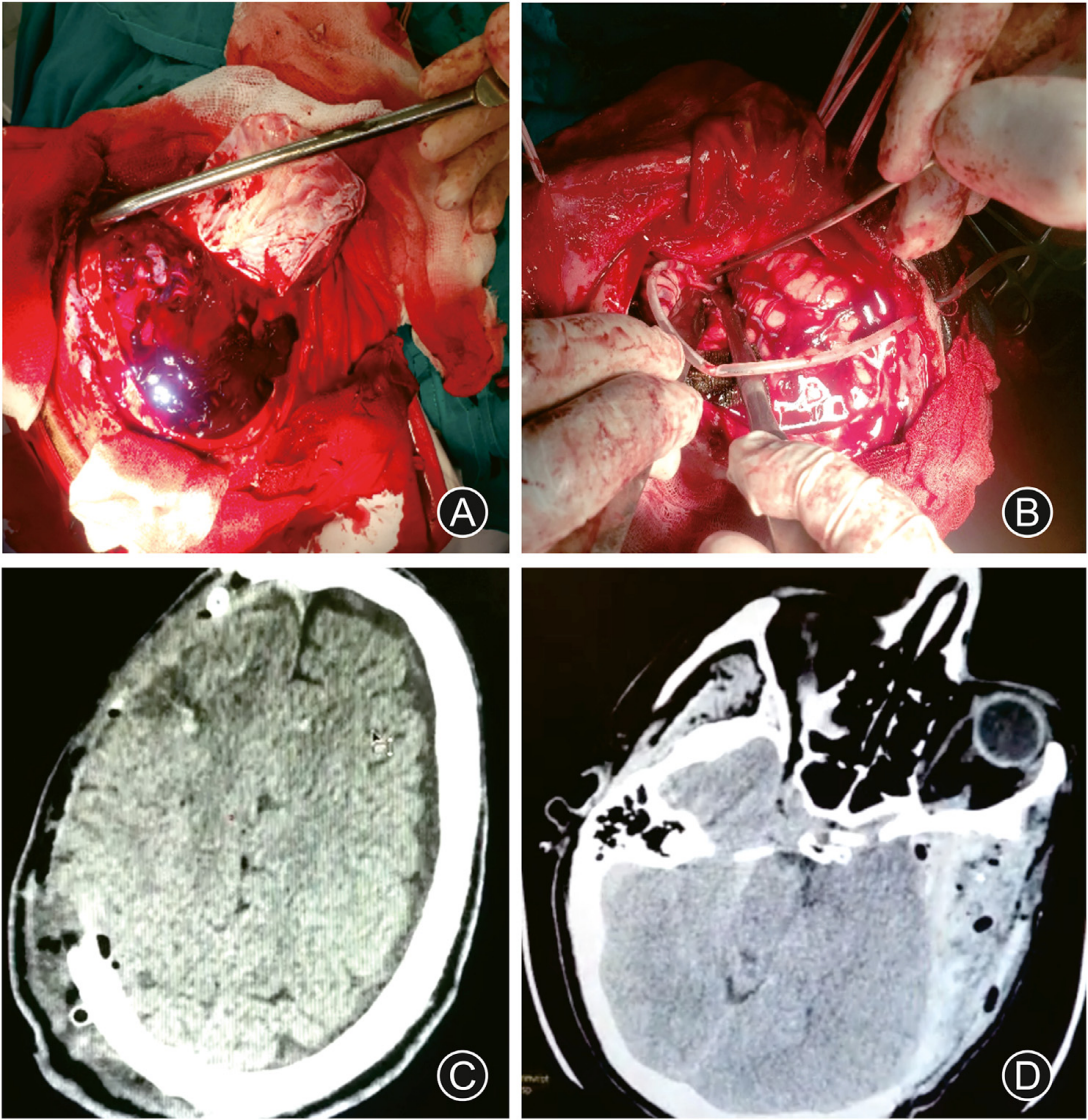
Intraoperative images and outcome. (A) After dural incision, the subdural blood clot covering the swollen and tight brain could be appreciated. (B) A temporary external drain was placed into the basal cistern, causing immediate brain relaxation. Postoperative axial CT showing the decompressive flap (C) and the drainage tube draining CSF from the basal cistern (D).

The patient underwent an emergency decompressive craniectomy on the right side. The minor wing of the sphenoid bone was drilled. The brain appeared swollen and tight. The dura was incised and the clot was evacuated. Following evacuation of the clot, the brain continued to swell (Fig. 2A).

We proceeded to perform a basal cisternostomy. First, we identified the optic nerve and opened the chiasmatic cistern and the carotid cistern. Supratentorial and infratentorial cisterns were separated by the arachnoid membrane of Liliequist. We incised the Liliequist membrane through the optico-carotid and lateral carotid corridors. A drain was placed through the Liliequist membrane (Fig. 2B).

A rapid outflow of CSF was observed. Copious irrigation with saline was performed. After few minutes, relaxation of the brain was observed. Dural suturing was performed and an epidural drainage was placed. Due to the open skull fracture and presence of foreign bodies, placement of the bone flap was avoided.

### Outcome and follow-up

Postsurgical axial CT showed a significant relaxation of the brain (Fig. 2C). The correct position of the drain in the basal cisterns was confirmed on an axial CT cut (Fig. 2D). The patient was monitored in the intensive care unit under sedation for 4 days. On postoperative day 5, the patient was transferred to the neurosurgical ward. On day 6, the patient had a GCS of 15 and the drain was removed. The patient was discharged on day 10 after admission with no neurological deficits.

## Discussion

Cisternal opening is a commonly used microsurgical technique to drain CSF, allowing easier access to skull base lesions. It has been proposed for the treatment of TBI only in recent years. The CSF-shift edema hypothesis may provide the fundamental rationale for evacuation of CSF from an edematous brain in traumatic injury.^6^ Previous research has proven that the brain parenchyma and basal cisterns communicate via the paravascular spaces, which is called Virchow-Robin spaces.^7^^,^^8^ According to the CSF-shift edema hypothesis, a subarachnoid hemorrhage following brain injury causes a high pressure gradient inside the cisterns, driving CSF into the brain parenchyma through the Virchow-Robin spaces. This results in raised intraparenchymal pressure and disruption of the glymphatic fluid system. Opening of the basal cisterns to atmospheric pressure reverses the CSF-shift through draining CSF from the brain parenchyma, achieving brain relaxation.^5^^,^^6^ In contrast, the conventional attempt to lower the intracranial pressure through a decompressive craniectomy alone fails to reduce the intraparenchymal pressure, allowing only for an outlet for the herniating brain.^9^

Further, previous research has identified a link between TBI and accumulation of metabolic wastes and misfolded proteins leading to long-term secondary brain damage.^10^ Basal cisternostomy may possibly help prevent secondary brain damage through early restorage of the glymphatic fluid circulation.^6^

Here, we presented the fast recovery of a patient who presented with a GCS of 6 and bilaterally fixed pupils. CT showed a midline shift, subdural hematoma and effaced basal cisterns. Therefore, according to the Traumatic Brain Injury Outcome Prediction Calculator, he had a 14-day mortality risk of 82.8% and an 85.7% chance of unfavorable outcomes at 6 months.^11^^,^^12^ We did not place the bone flap back. Previous reports, however, have shown an immediate floating bone flap replacement to be feasible and safe.^4^

A limitation of this procedure is that the basal cisternostomy can only be performed using microsurgical techniques. It thus requires specialized training and a round-the-clock availability of microscopic facilities which pose a challenge to many centers around the world.^13^

In conclusion, basal cisternostomy is a safe and feasible microsurgical procedure for the management of TBI. It can effectively evacuate CSF and relax the brain. Compared to decompressive craniectomy, basal cisternostomy depends on the use of microsurgical techniques. Nonetheless, it may alleviate the economic and social burden caused by TBI. Large scale clinical trials are needed to support the published data and encourage more widespread acceptance of the approach.

## Funding

Nil.

## Ethical statement

This case report was completed in accordance with the Declaration of Helsinki as revised in 2013. No copyright material was used in this manuscript. This work has never been published before and is currently not being considered for publication in any other journal.

## Declaration of competing interest

No author has competing interests to declare.

## Author contributions

According to CRediT author statement guidelines, author contributions are listed as below:

Conceptualization: Manuel De Jesus Encarnacion Ramirez, Ibrahim E. Efe;

Investigation: Manuel De Jesus Encarnacion Ramirez, Rossi Evelyn Barrientos Castillo, Anton Vorobiev, Nikita Kiselev;

Visualization: Manuel De Jesus Encarnacion Ramirez, Ibrahim E. Efe; Writing – Original Draft: Manuel De Jesus Encarnacion Ramirez, Amaya Alvarez Aquino; Writing – Review & Editing: Ibrahim E. Efe;

Supervision: Ibrahim E. Efe; Project Administration: Manuel De Jesus Encarnacion Ramirez.
